# Synthesis, Properties and Applications of Germanium Chalcogenides

**DOI:** 10.3390/nano12172925

**Published:** 2022-08-25

**Authors:** Stefania M. S. Privitera

**Affiliations:** Institute for Microelectronic and Microsystems (IMM), National Research Council (CNR), Zona Industriale Ottava Strada 5, 95121 Catania, Italy; stefania.privitera@imm.cnr.it

Germanium (Ge) chalcogenides are characterized by unique properties which make these materials interesting for a very wide range of applications, from phase change memories to ovonic threshold switches, from photonics to thermoelectric and photovoltaic devices. In many cases, physical properties can be finely tuned by doping or by changing the Ge amount, which can thus play a key role in determining the applications, performance, and even the reliability of the devices. In this Special Issue, we include 11 articles, mainly focusing on applications of Ge chalcogenides for nonvolatile memories.

Most of the papers were produced with funding received from the European Union’s Horizon 2020 Research and Innovation program under grant agreement n. 824957 for the project “BeforeHand: Boosting Performance of phase change Devices by Hetero- and Nanostructure Material Design”.

Two contributions [1,2] are related to the prototypical Ge_2_Sb_2_Te_5_ compound, which is a widely studied composition and is already integrated in many devices such as optical and electronic memories. In [1], M. Bertelli at al. report on the structural and electrical properties of GST225 grown on polyimide, a flexible substrate whose use could enable novel applications in the market of electronics, for example, flexible nonvolatile memories for the IoT, or smart sensors for food and drug monitoring. The paper reports information about the layer evolution during amorphous-to-cubic and cubic-to-trigonal transitions, and the related electrical contrast.

In [2], M. A. Luong et al. investigate the atomistic mechanisms related to nitrogen doping, which is known to improve some key characteristics of the materials, such as the amorphous stability and the resistance drift. These effects are ascribed to the increased viscosity of the N-doped amorphous state and to the reduced diffusivity resulting from the formation of N-Ge bonds, demonstrating that the origin of the effect of N on crystallization is attributed to the ability of N to bind to Ge in the amorphous and crystalline phases and to unbind and rebind with Ge along the diffusion path during annealing.

Another approach to improve the thermal stability of the amorphous phase is presented in [3], where starting from the GeTe alloy, X. Wang and coauthors incorporate indium, obtaining three typical compositions in the InTe-GeTe tie line, and propose a chemical composition with both improved thermal stability and sizable optical contrast for photonic applications.

Ge-rich GeSbTe (GST) alloys are currently explored for embedded memory applications, with the aim to increase the crystallization temperature, therefore improving the amorphous phase stability. However, deposited homogenous alloys are thermodynamically unstable and undergo phase separation upon annealing.

Five articles of this Special Issue focus on Ge-rich GST alloys, exploring their electronic and electrical properties [4,5,6,7] as well as decomposition pathways, including from a theoretical point of view [8].

In [4], S. Cecchi et al. identify some possible routes to limit Ge segregation, investigating Ge-GST compositions deposited by molecular beam epitaxy in the amorphous phase with low or high (>40%) amounts of Ge. Electrical resistance and phase formation are studied upon annealing up to 300 °C.

In [5], A. Diaz Fattorini and coauthors deposit Ge-rich GST with a composition of Ge_29_Sb_20_Te_28_ via physical vapour deposition (PVD). They study the electronic properties and phase formation and report the electrical characterization of a single memory cell, showing the possibility to enhance the thermal stability up to 230 °C while maintaining a fair alignment of electrical parameters with the current state of the art of conventional GST alloys.

The contribution of D. Tadesse Yimam et al. [6] investigates the phase separation of GST523 into multiple phases in melt quenched bulk and annealed thin films, identifying the formation of GST123 and GST324 alloys in all length scales.

The alloy compositions and the observed phase separation pathways reported in [4,6] agree to a large extent with the theoretical results from the density functional theory calculations, as presented in [8], where O. Abou El Kheir and M. Bernasconi perform high-throughput calculations to uncover the most favorable decomposition pathways of Ge-rich GST alloys. They also construct a map of decomposition propensity, suggesting a possible strategy to minimize phase separation while still maintaining a high crystallization temperature.

In [7], A. Kumar and coauthors investigate the effect of Ge-rich GST in nanowires self-assembled through the vapor–liquid–solid mechanism. Both Ge-rich GST core and Ge-rich GST/Sb_2_Te_3_ core shells are extensively characterized with several techniques to analyze the surface morphology, crystalline structure, vibrational properties and elemental composition.

Other tree contributions [9,10,11] are focused on the effect of the interfaces, since in nanomaterials, element interdiffusion at the interfaces represents a crucial factor.

In [9], V. Bragaglia et al. investigate this aspect in projected phase change memories, in which the storage mechanism is decoupled from the information retrieval process via a projection liner. The interface resistance between the phase change chalcogenide material and the projection liner is an important parameter, and therefore a metrology framework is established to assess the quality of the interfaces through X-ray reflectivity, X-ray diffraction, and transmission electron microscopy.

As another important case in which interfaces play a significant role, article [10] by C. Chèze and coauthors reports the full characterization of the electronic properties of double-layered heterostructures made by Ge-rich GST deposited by PVD on Sb_2_Te_3_ and on Ge_2_Sb_2_Te_5_. Information on interdiffusion and on the evolution of the composition across the interface was obtained; it was found that, in both heterostructures, the final composition was GST212, which is a thermodynamically favorable off-stoichiometry alloy in the Sb-GeTe pseudo-binary line.

The interdiffusion at the interface of core–shell nanowires with a Sb_2_Te_3_ shell over GeTe and a Ge-rich GST core is studied in [11] by Kumar et al. by examining the morphological and structural characteristics. No elemental interdiffusion between core and shell is revealed, suggesting that their structural phases can change independently based on alloy compositions, thus demonstrating a straightforward method to provide core–shell nanowire heterostructures formed by two-phase chalcogenide materials with different crystallization temperatures and switching speeds.
